# Microwave Frequency Doubler with Improved Stabilization of Output Power

**DOI:** 10.3390/s23073598

**Published:** 2023-03-30

**Authors:** Piotr Kwiatkowski, Michał Knioła, Zenon Szczepaniak

**Affiliations:** Faculty of Electronics, Military University of Technology, 00-908 Warsaw, Poland

**Keywords:** frequency doubler, multiplier, power stabilization, frequency synthesis, upconversion

## Abstract

The passive multipliers based on semiconductor diodes, most frequently a Schottky type, should be driven by a certain value of input power, where the conversion losses are optimal. This means that the variation in the input power level causes the change in the output power level. A solution to this issue is the integration of an output power amplifier, which in the state of saturation provides quasi-stabilization of the output power. Practically, this approach gives an unsatisfactory performance: weak stabilization or narrow input power range. This paper comprises a concept of an active frequency multiplier with the use of one FET transistor and a special adaptive bias circuit in order to obtain a very wide input power range when the output power is stable. The principle of the operation, design guidelines, and measurement results have been presented for an example circuit of the frequency doubler. The results show the possibility to obtain up to 10 dB input power range for a 1 dB change in output power level without the use of additional amplifiers.

## 1. Introduction

In numerous signal synthesizer circuits, there is a need to create a signal that has a stable output power, especially within a given frequency band. The issue of power stabilization is most important, especially when the frequency upconversion of a signal with variable power level is concerned. In such a situation the output signal from a frequency mixer, i.e., the upper sideband is directly related to the power at IF (intermediate frequency) input of a mixer. This issue is also very important in the case where a synthesizer contains cascaded frequency multipliers. Frequency multipliers have increased sensibility of output power at a given harmonic when the input power varies. This is because the output signal is a nonlinear product of N-th order for one-tone input excitation [1]. Then, the output power vs. input power slope is equal to N when logarithmic notation is used. The additional issue is the temperature dependence of power gain in transistor amplifiers. All these things considered together portray the vital need to obtain stable power at the output of frequency conversion circuit in case of variable input power.

In general, there are two methods to fulfil this requirement. The first one is the use of a special limiting circuit called a power leveler, which is a kind of power limiter driven into output power saturation range. Depending on the circuit-level solution chosen (passive—diodes, active—transistors) the output power slope may be equal from a fraction of dB to 1–2 dB for a 3 dB input power change [2,3,4,5,6,7,8,9,10,11]. Such a circuit may be used at the end of a signal synthesis cascade.

The other method is the stabilization of output power variations for every frequency multiplier used in the cascade or circuit. The most common method, implemented in microwave circuits of known microwave components producers, e.g., Minicircuits, is cascading the passive frequency multiplier with an output transistor amplifier.

**Figure 1:**
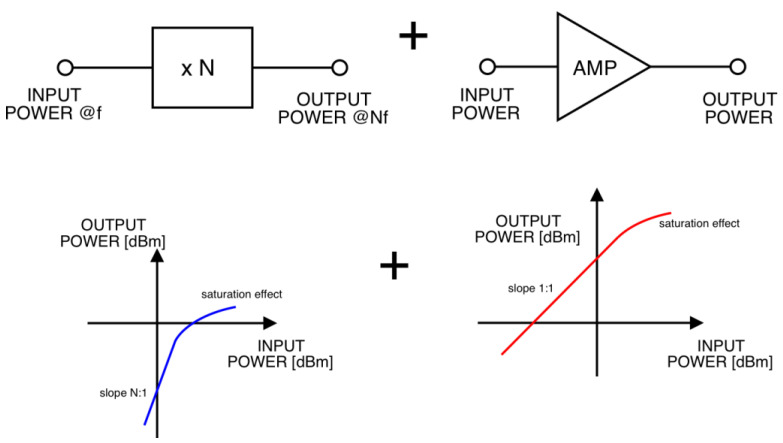
Block diagram of frequency multiplier with output amplifier.

This method relies on two nonlinear phenomena. First is the saturation of output power versus input power in a frequency multiplier for higher input power values. This effect gives a kind of power limitation and hence quasi-stabilization; however, the slope is lower than N:1 but still not satisfying. Moreover, in the saturation range, a frequency multiplier presents lowered conversion efficiency. The second nonlinear effect is the saturation of the output power of the transistor amplifier. This amplifier is used here i.e., in this cascade for two reasons: to amplify the multiplier output power and to introduce additional power limiting capability. The combination of these two limiting actions gives better output power stabilization at the cost of complexity and power consumption in the cascade (Figure 1).

In some realizations, this solution offers a still unsatisfying output power stabilization. As an example, the XX1000-QT (Minicircuits) frequency multiplier may be pointed out. Here, the output power slope is about 1 (5 dB/5 dB). However, the device XX1002-QH (Minicircuits) offers very good stability of output power level (0.2 dB) but for a relatively small input power range of 6 dB. More improved results are presented in the paper [12], i.e., the output power stabilization within 1 dB for up to 8 dB of input power range, but at the cost of extended complexity of the circuit. The circuit contains a driving amplifier, two diodes in balanced topology, and an output amplifier with three stages.

Another approach to frequency multiplying is the use of an active circuit based on a transistor, most frequently a type of Field Effect Transistor (FET). The examples of single FET-based frequency multipliers optimized for best conversion efficiency and without optimization of output power stability are presented in the papers [13,14,15,16]. In [13], there is a single FET multiplier-type mixer for an input frequency of 2 GHz. In [14], there is a single FET active multiplier without stabilization (1 dB/1 dB) with an input frequency of 1.4–1.6 GHz and with a gate-source voltage of constant value equal to 0 V. An example of a frequency doubler for high power applications is shown in [15]. Here, the input frequency is about 2 GHz and the output power stability is 5 dB for 15 dB of the input power range. In the paper [16], there is a single FET doubler for 4–8 GHz input frequency with analysis of optimal gate-source voltage for the maximum output power for variable input power value. Active FET-based multipliers are also used for very high frequencies, as the examples may serve papers [17,18]. In [17], the one stage doubler for 76 GHz output frequency without stabilization but optimized high gain is shown. In the paper [18], a frequency doubler with two FET transistors is presented. The circuit has an input frequency of 14 GHz and an output power stabilization of about 1 dB for 3 dB of input power range (estimated from conversion gain).

The improvement of output power stabilization may be achieved by the use of more than one transistor in an active multiplier. In the paper [19], there are two FETs, one in class C as the multiplier stage and one in class B as the output amplifier. The results are 1 dB of output power stability for the input power range of 5 dB. In the paper [20], the use of two transistors gives the result of 1 dB of output power stability for the input power range of 7 dB.

In the paper [21], there is a multiplier circuit with 4 FETs and an output amplifier with three FET transistors. The results are 1 dB of output power stability for the input power range of 5 dB. Moderate and improved stability range is shown in papers [22] and [23]. The results are 1 dB of output power stability for the input power range of 4 dB in [22] and about 2.5 dB for 15 dB in [23]—that is the best result for a single device found in the literature.

Concerning the issue of output power stabilization in frequency multipliers, the authors propose a new approach for designing active frequency multipliers.

This approach relies on the use of a single-stage FET transistor frequency multiplier with the self-bias of gate-source junction. Self-bias means the possibility of changing the bias voltage according to the temporary value of the input power, this kind of technique is used in circuits containing diodes [24]. The multiplier circuit presented in the paper is specially optimized in order to obtain the output power at given harmonics as stable as possible for variable input power, with the use of only one semiconductor device.

## 2. Materials and Methods

In order to investigate the possibility of achieving frequency multiplication with the increased stability of output power an active FET-based frequency doubler has been analyzed. The initial assumptions have been as follows:

- Frequency multiplication order equal to N = 2;
- Input frequency f = 1 GHz;
- Stabilization of output power at second harmonics (2 GHz) within 1 dB range for at least 6 dB input power range (generally, as wide as possible).

The methodology of investigations consists of several steps:

- Concept of the circuit;
- Defining of the nonlinear model describing the circuit’s work;
- Numerical calculations of output power;
- Microwave design and nonlinear simulations with the use of AWR Microwave Office;
- Circuit realization and measurements.

### 2.1. Concept

**Figure 2:**
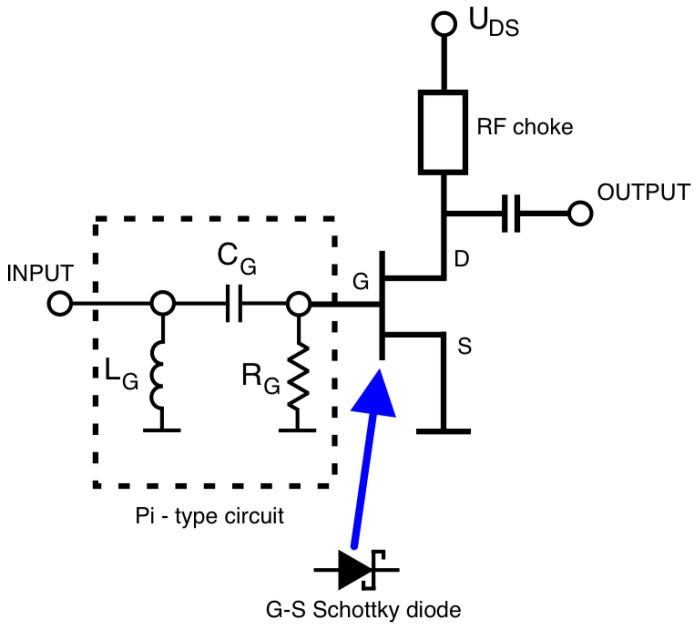
Diagram of the proposed frequency doubler.

The general scheme of the proposed frequency multiplier has been shown in Figure 2. It assumes the use of a microwave FET transistor in the common-source configuration. The drain-source voltage is applied to the drain terminal and the gate-source voltage is initially set to zero volts with the use of a resistor connected between the gate terminal and the common ground. The key property is the use of a pi-type RLC circuit consisting of inductor L_G_, capacitor C_G,_ and resistor R_G_. This circuit is connected to the gate terminal and together with the gate-source (G-S) Schottky junction forms a kind of amplitude detector. When the input power is equal to zero the G-S bias voltage is also equal to zero volts. Further, when the input power is increased the G-S voltage changes and is equal to the detected value of input power. The polarity of the detected voltage causes the G-S junction to be biased in reverse. Therefore, the more input power is applied the bias point of the transistor is more shifted toward the cut-off, and further below the value of the cut-off.

This situation causes clipping of the output signal, and finally, the output current waveform contains harmonics of the input frequency.

### 2.2. Modeling and Calculations

**Figure 3:**
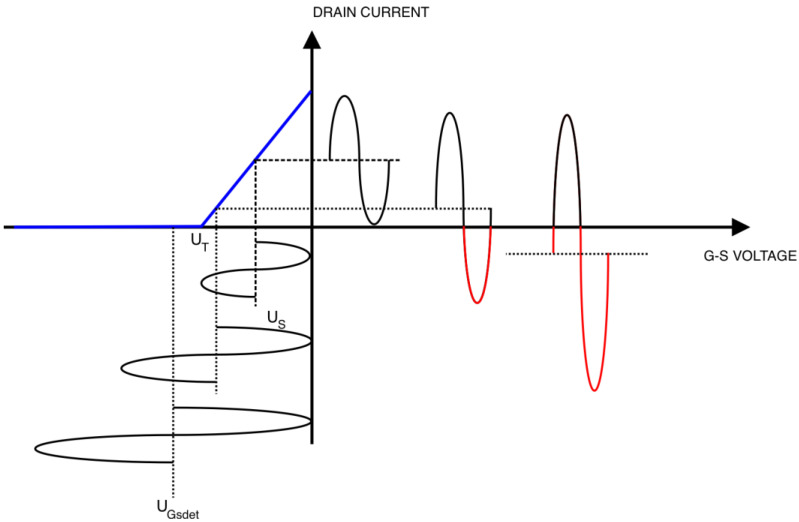
Working principle of the proposed circuit.

The principle of the operation pointed out in the previous section has been illustrated in Figure 3. The simplified model shown in this figure consists of a simplified (piecewise linear) transient characteristic of drain current versus gate-source voltage U_GS_ and superposition of bias points with the input voltage waveforms. The drain current saturation effect for U_GS_ > 0 V has been omitted for simplicity.

This simplest model is sufficient to present and further calculate the key effects existing in this kind of circuit.

Further analysis was carried out assuming the initial value of the U_GS_ voltage equal to zero and the excitation of the input of the limiter system with a sinusoidal signal with the amplitude U_S_. 

For gate-source voltages U_GS_ greater than the cut-off voltage U_T_, the drain current can be approximated by a linear relationship:

(1) $$I_{D}=\frac{-I_{D0}}{U_{T}}\cdot U_{\mathrm{GS}}+I_{D0}=a\cdot U_{\mathrm{GS}}+b,$$

where

I_D0_—value of drain current for gate-source voltage equal to zero,
U_T_—transistor threshold voltage.

Initially, for very small values of the amplitude U_S_, the detected voltage U_GSdet_ has a negligibly low value and can be assumed to be zero. The circuit is in the linear operating range. 

An increase in the amplitude of the control voltage U_S_ causes the appearance of a non-zero value of the detected voltage U_GSdet_ and shifts the operating point of the transistor.

The detected voltage U_GSdet_ can be approximated by a linear function:

(2) $$U_{\mathrm{GSdet}}=c\cdot U_{S},$$

where

c—detector coefficient [V/V].

The linear operation range is maintained until the minimum momentary driving voltage equals the value of the cut-off voltage U_T_.

(3) $$U_{T}=-U_{S}+U_{\mathrm{GSdet},}$$

For larger values of the driving voltage amplitude U_S_, the sinusoidal waveform of the drain current is clipped unilaterally, this effect becomes stronger as U_S_ increases until the drain current waveform consists only of a series of pulses corresponding to the truncated peaks of the sinusoidal waveform. This situation is shown in Figure 3, where the red part of the drain current waveform is clipped out.

The condition for non-linear range can be written by substituting Equation (2) to (3):

(4) $$U_{\mathrm{snlin}}>\frac{-U_{T}}{1-c,}$$

where

U_snlin_—the value of the driving voltage amplitude for the non-linear range.

As has been stated before, the output current waveform is clipped. For the clipped sine waveform of the drain current, the harmonic content for n-th order may be found with the use of relationships (5) and (6) [25]:

(5) $$I_{\mathrm{dn}}=\frac{2I_{p}}{\pi }f{\left(\phi \right)}_{n},$$

(6) $$f{\left(\phi \right)}_{n}=\frac{\sin\left(n\phi \right)\cos\left(\pi \right)-\mathrm{nsin}\left(\phi \right)\cos\left(n\phi \right)}{n\left(n^{2}-1\right)\left(1-\cos\left(\phi \right)\right)},$$

For the circuit from Figure 3, the variables current amplitude I_p_ and conduction angle ϕ may be expressed as:

(7) $$I_{p}=a\cdot \left(U_{\mathrm{GSdet}}+U_{s}\right)+b,$$

(8) $$\cos\varphi =\frac{U_{T}-U_{\mathrm{GSdet}}}{U_{s}},$$

Substituting for the frequency doubler n = 2 one may obtain the function of conduction angle in a form:

(9) $$f{\left(\phi \right)}_{2}=\frac{\sin\left(2\phi \right)\cos\left(\pi \right)-2\sin\left(\phi \right)\cos\left(2\phi \right)}{2\left(2^{2}-1\right)\left(1-\cos\left(\phi \right)\right)},$$

The set of Equations (1)–(9) allows us to perform calculations of the output power at the second harmonic versus input power level. For the following calculations, it was assumed that the input and output powers are referenced to standard 50 Ω load impedances.

The results of these simulations are shown in the following graphs showing the output power of the second harmonic as a function of the input power, as well as the impact of changes (selection) of the parameters: cut-off voltage U_T_ and detection coefficient c. The U_T_ parameter is closely related to a specific FET transistor and its value cannot be changed, while the detection constant c can be “adjusted” by changing the resistance value of the R_G_ resistor from Figure 2.

For investigations the following two cases have been calculated:

- Constant detector coefficient c and variable threshold voltage U_T_;
- Constant threshold voltage U_T_ and variable detector coefficient c.

**Figure 4:**
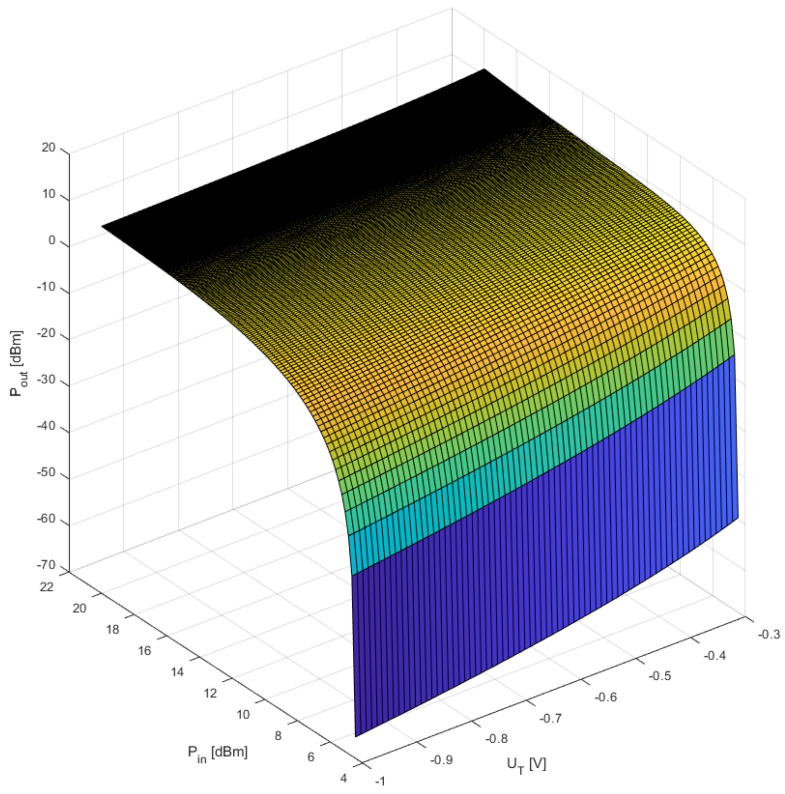
The impact of changes in the value of the U_T_ parameter at a constant value of the c = −0.9 V/V parameter on the range of output power stabilization.

**Figure 5:**
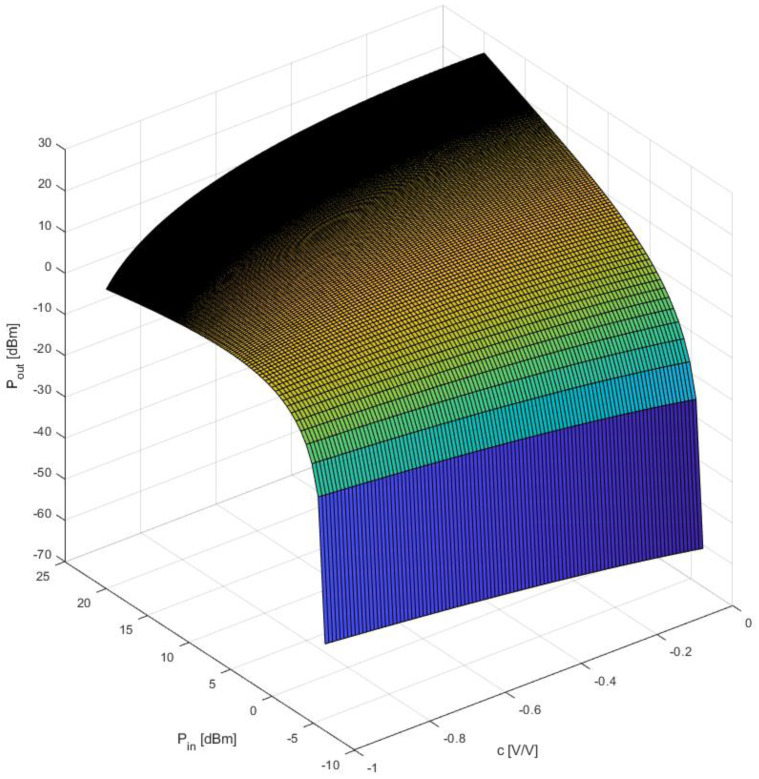
The impact of changes in the value of the detection constant c at a constant value of the U_T_ = −0.3 V parameter on the “flatness” of the output power at the second harmonic.

The results of both simulations are shown in Figure 4 and Figure 5. As can be seen in the figures below, there is a strong dependance on the output power for various values of the parameters c, U_T,_ and input power. When the value of the detection coefficient is changed toward a value of −1 the output power at the second harmonic becomes more stable within a certain range of input power.

The optimal behavior of the output power stabilization mechanism for the second harmonic appears for the values of the U_T_ parameters and the detection constant c for such values as: U_T_ = −0.3 V and c = −0.9 V/V.

### 2.3. Circuit Design

**Figure 6:**
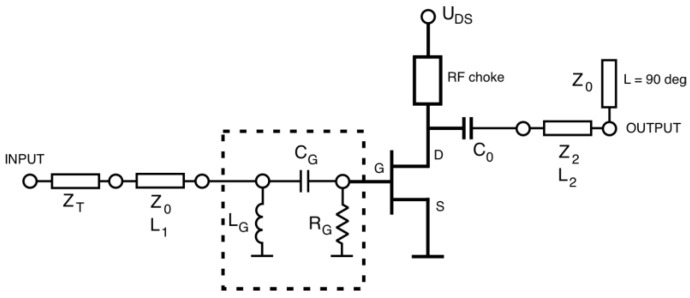
Frequency doubler circuit with ideal components.

The first step of the real circuit design is the definition and implementation of an ideal-case version of the circuit. This circuit is shown in Figure 6.

This consists of an ATF-36163 PHMET and auxiliary detector circuits L_G_, R_G_, and C_G_ as the key components, together with additional circuits:

1. Input matching formed with two transmission lines, first of Z_0_ = 50 Ω characteristic impedance and adjustable length of L_1_. The second line is the quarter-wave transformer with a length of 90° and adjustable characteristic impedance Z_T_.

2. Output matching circuit. Here, this circuit is assumed to have the simplest form of one section of transmission line with adjustable characteristic impedance and length. This circuit also influences the phase of the fundamental frequency signal returning into an active part of the multiplier after reflection caused by a quarter-wave open-end stub.

3. Fundamental frequency rejection circuit at the output of the whole circuit. Here, a quarter-wave open-end stub at the fundamental frequency is used.

**Figure 7:**
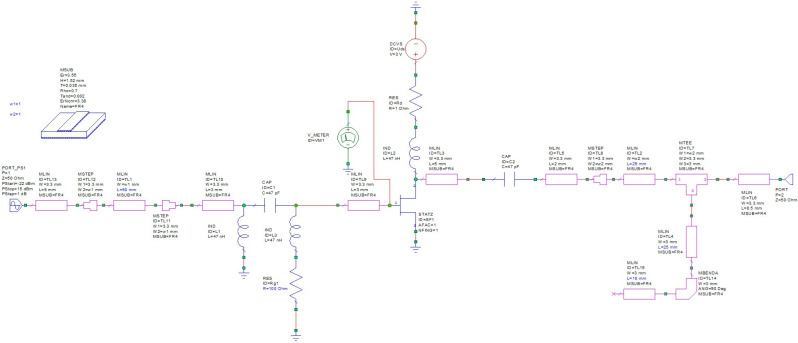
Frequency doubler circuit implemented in Microwave Office with realistic components.

The second step is the implementation of the real parameters of the circuit components, e.g., transmission lines in the form of microstrip lines at a given substrate—Figure 7. For the purposes of the design and simulation, the AWR Microwave Office simulator has been used. However, one may use any CAD simulator with the Harmonic Balance engine.

The final optimization of the circuit gave the values of components parameters as well as the prediction of the input power range for output power stabilization and output power ripple.

The parameters are as follows:

- C_G_ = 47 pF, R_G_ = 100 Ω, L_G_ = 47 nH;
- Z_T_ = 150 Ω, L_1_ = 3 mm (FR-4 laminate), L_2_ = 29 mm (FR-4 laminate);
- U_DS_ = 3 V.

**Figure 8:**
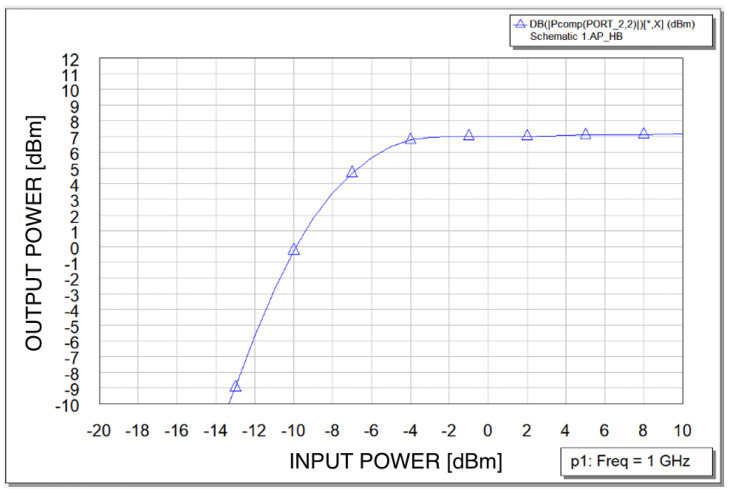
Output power at 2 GHz (second harmonic) versus input power at 1GHz (fundamental) for the frequency doubler simulated in Microwave Office.

An example of simulation results is shown in Figure 8.

## 3. Results

**Figure 9:**
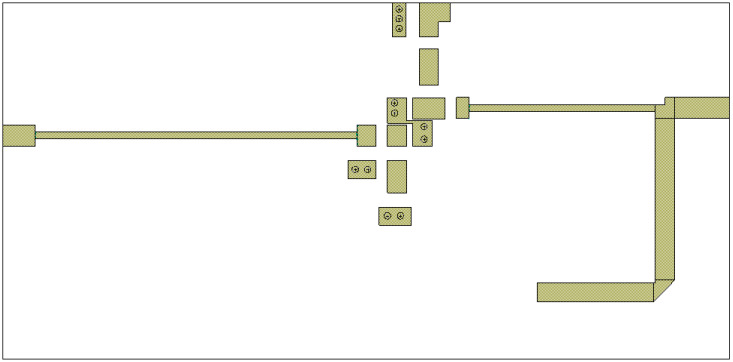
Layout of the designed frequency doubler circuit.

**Figure 10:**
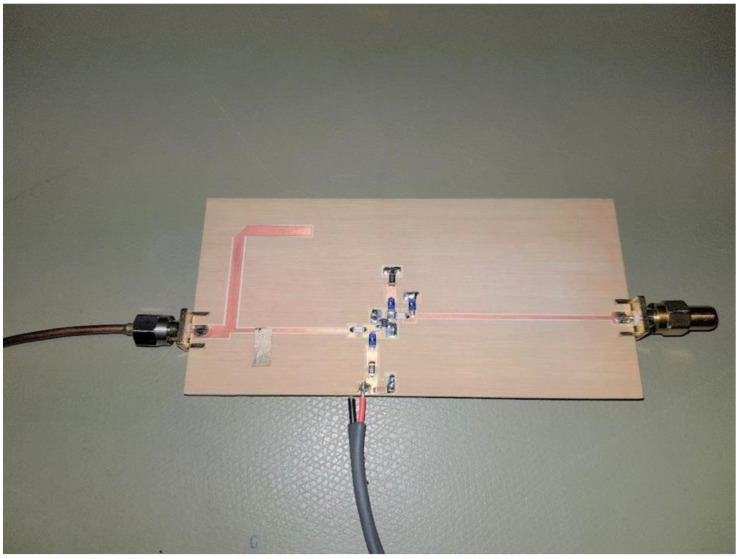
Picture of manufactured frequency doubler circuit.

The designed frequency doubler has been fabricated on FR-4 glass-epoxy laminate. A circuit layout and a picture of the ready circuit are shown in Figure 9 and Figure 10.

**Figure 11:**
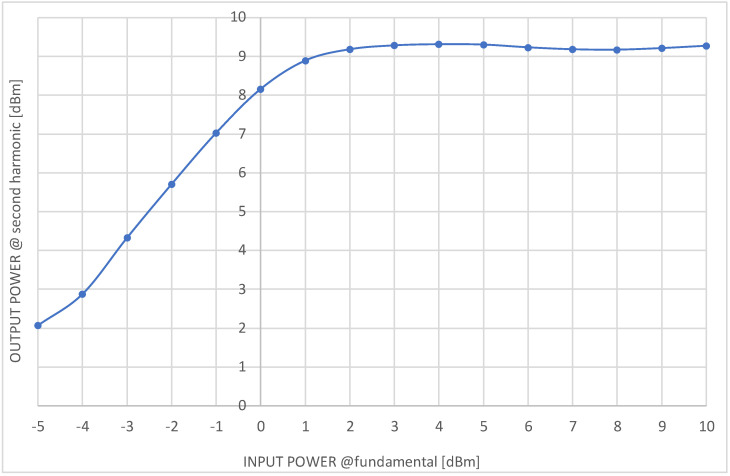
Measurements results of output power at 2 GHz (second harmonic) versus input power at 1 GHz (fundamental) for manufactured frequency doubler.

The circuit was assembled and initially checked in order to ensure that there is a proper bias supply and lack of any self-oscillations. Next, measurement investigations were conducted. The measurement stand consisted of a signal generator and a spectrum analyzer. All the coaxial lines, connections, and adapters were calibrated initially, i.e., all the additional losses were measured. The final measurement results of the output power at the second harmonic versus input power level at a fundamental frequency equal to 1 GHz (generator available power) are presented in Table 1 and Figure 11. The input power is referred to as the doubler input port and the output power is referred to as the circuit’s output port.

## 4. Discussion

The frequency doubler presented in the paper offers very good output power stability—Figure 11. The cost of this feature is a dedicated circuit design and optimization. For the presented circuit, the input power range when the output power is stable within 1 dB of change equals about 10 dB (9.8 dB). The circuit in the form presented here produces other harmonics of the input signal frequency. Therefore, for further use in frequency synthesizers an output bandpass filter is needed.

The circuit offers conversion gain of 8 dB to −0.7 dB for input power range from 0 to 10 dBm, respectively, when the output power is stabilized.

This is a very good result compared to a kind of passive diode doubler with an additional output amplifier. In such a reference solution, the amplifier works in the A class and amplifies doubler output power, apparently lowering conversion losses. In comparison, the doubler presented in the paper works with the variable bias point being shifted from the B class to the C class at higher input power levels when actual stabilization occurs. This effect causes low bias power consumption. 

The range of stabilization of the output power as a function of the input power differs between the circuit designed in the Microwave Office and the one made in practice. This is due to many factors affecting the obtained output power values, including the accuracy of the PCB manufacturing, the accuracy of the assembly of SMD components, the connections to the ground plane of the board, as well as the impact of the actual value of the threshold voltage of the FET transistor, which may differ from that assumed in the transistor model.

For a multiplier, it is difficult to apply the S-matrix notation for output matching because of the fact that consideration of the output (here S_22_) scattering parameter must be performed with simultaneous excitation of the input port at the fundamental frequency. This is contrary to the assumption of no incoming waves into the circuit when reflection coefficients are defined. Therefore, the output “matching” circuit should allow obtaining the best output power extraction when the excitation signal is present at the input port.

## 5. Conclusions

The paper comprises an analysis and explanation of the nonlinear behavior of transistor-based frequency doublers. The design and measurement results of the circuit have been presented. The doubler circuit described in the paper has been designed for CW (continuous wave) input signal at 1 GHz. This specific requirement results from a project, that the authors were involved in. For a doubler with a specified wider input frequency range (not CW), the working principle and design methodology is the same. The design will be different because of the need for input and output matching and output filtering, which work within specified frequency band. However, the issues of optimization of the input detector circuit and input/output transforming circuits are still the same.

The obtained results, i.e., the input power range of approx. 10 dB for stabilization of the output power at second harmonics within 1 dB change is a very good result compared to solutions described in the Introduction section, considering the doubler consists of only one semiconductor element. There is no need for additional semiconductor diodes or amplifiers.

The methodology presented in this paper may be further applied to a design and analysis of other kinds of frequency multipliers, e.g., triplers or quadruplers. The measure of stabilization quality defined as the input power range for 1 dB change of output power level seems to be convenient and useful for other circuit evaluations.

## Figures and Tables

**Table 1:** Measurements results of output power at 2 GHz (second harmonic) versus input power at 1 GHz (fundamental) for manufactured frequency doubler.

Pin (dBm)	Pout (dBm)
−20	−29.29
−14	−16.92
−10	−7.52
−5	2.07
−4	2.88
−3	4.33
−2	5.71
−1	7.03
0	8.16
1	8.89
2	9.18
3	9.28
4	9.31
5	9.30
6	9.23
7	9.18
8	9.17
9	9.21
10	9.27

## Data Availability

The data are not publicly available due to privacy restrictions.
